# High-performance light-driven heterogeneous CO_2_ catalysis with near-unity selectivity on metal phosphides

**DOI:** 10.1038/s41467-020-18943-2

**Published:** 2020-10-13

**Authors:** Yang-Fan Xu, Paul N. Duchesne, Lu Wang, Alexandra Tavasoli, Feysal M. Ali, Meikun Xia, Jin-Feng Liao, Dai-Bin Kuang, Geoffrey A. Ozin

**Affiliations:** 1 Materials Chemistry and Nanochemistry Research Group, Solar Fuels Cluster, Department of Chemistry, University of Toronto, Toronto, Ontario M5S 3H6 Canada; 2 MOE Key Laboratory of Bioinorganic and Synthetic Chemistry, Lehn Institute of Functional Materials, School of Chemistry, Sun Yat-sen University, 510275 Guangzhou, Guangdong P. R. China

**Keywords:** Heterogeneous catalysis, Photocatalysis, Nanoparticles

## Abstract

Akin to single-site homogeneous catalysis, a long sought-after goal is to achieve reaction site precision in heterogeneous catalysis for chemical control over patterns of activity, selectivity and stability. Herein, we report on metal phosphides as a class of material capable of realizing these attributes and unlock their potential in solar-driven CO_2_ hydrogenation. Selected as an archetype, Ni_12_P_5_ affords a structure based upon highly dispersed nickel nanoclusters integrated into a phosphorus lattice that harvest light intensely across the entire solar spectral range. Motivated by its panchromatic absorption and unique linearly bonded nickel-carbonyl-dominated reaction route, Ni_12_P_5_ is found to be a photothermal catalyst for the reverse water gas shift reaction, offering a CO production rate of 960 ± 12 mmol g_cat_^−1^ h^−1^, near 100% selectivity and long-term stability. Successful extension of this idea to Co_2_P analogs implies that metal phosphide materials are poised as a universal platform for high-rate and highly selective photothermal CO_2_ catalysis.

## Abstract (web summary)

There exists an urgent need to develop new materials to convert CO_2_ to useful products. Here, authors demonstrate metal phosphide nanoparticles to enable light-driven CO_2_ hydrogenation with high activities and near-unity selectivity.

## Introduction

In the field of heterogeneous catalysis, gaseous or liquid reactants undergo chemical reactions on the surface of a solid material. These reactions can be enabled thermochemically, electrochemically, biochemically or photochemically^1–8^. The surface reactivity of catalytically active sites is typically discussed in terms of electronic, geometric and support effects^9–15^. Moreover, as the number of catalytically active sites scales with the surface area of the catalyst, size matters, especially at the nanoscale. Yet most heterogeneous catalysts exhibit surface structure heterogeneity that depend upon particle size and exposed facets, which influences reactivity and selectivity patterns. Furthermore, the size distribution and facet exposure can change over the course of a catalytic reaction, having a deleterious effect on selectivity patterns and long-term stability. Ideally, a heterogeneous catalyst comprised of single-size, high surface area nanoparticles, with uniform shape and facet exposure that remains stable under reaction conditions could overcome these problems; in practice however, they have rarely been achieved.

Metal phosphides form a class of solids that provide the envisioned atomic and crystalline perfection with a structure based upon highly dispersed metal nanoclusters chemically integrated in a P lattice. These materials abound with diverse stoichiometry across a wide range of metals in the periodic table offering opportunities as catalysts for electrochemical hydrogen evolution reactions, hydro-processing, and so forth^16–20^. However, their appealing potential for heterogeneous (photo)catalytic CO_2_ hydrogenation remains to be unlocked.

Herein, we focus attention on an archetypal nickel phosphide, Ni_12_P_5_, with a unique surface structure based upon well-separated few-atom Ni nanoclusters. This structure allows Ni_12_P_5_ to function as an exceptionally active, selective, and stable heterogeneous catalyst for the photothermal reverse water gas shift (RWGS) reaction under light stimulation. Moreover, this concept is further extended to the construction of Co_2_P analogs, thus revealing a bright future for the earth-abundant transition metal phosphides as a class of heterogeneous catalysts for CO_2_ hydrogenation application. This potential is demonstrated by the high conversion rates and selectivity and long-term stability observed for these transition metal phosphides in heterogeneous CO_2_ hydrogenation reactions driven by solar energy.

## Results

### Material synthesis and characterizations

**Fig. 1:**
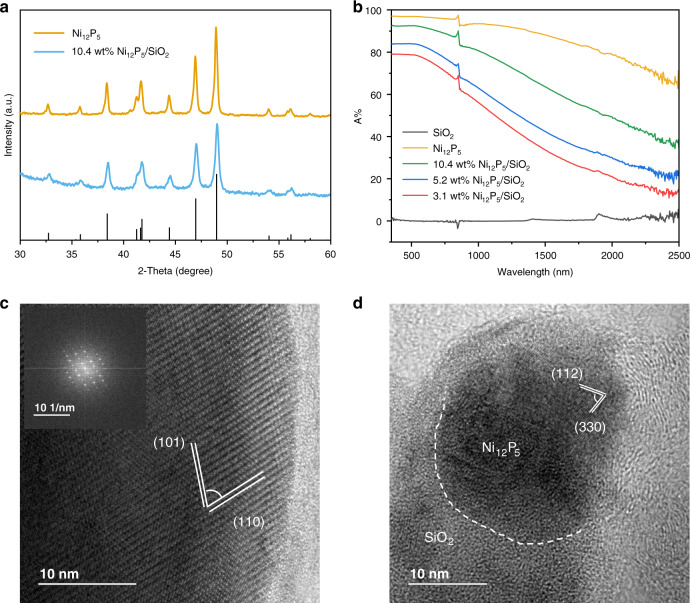
Characterization of Ni_12_P_5_ nanoparticles and the 10.4 wt% Ni_12_P_5_/SiO_2_ nanocomposite. **a** XRD patterns, black lines indicate the standard peaks of tetragonal Ni_12_P_5_ (PDF#74-1381). **b** UV-vis-NIR spectra of Ni_12_P_5_, SiO_2_ and Ni_12_P_5_/SiO_2_ samples loaded onto a binder-free borosilicate glass microfiber filter support (0.5 mg cm^−2^). **c** HRTEM image of Ni_12_P_5_, inset is the corresponding FFT electron diffraction pattern. **d** HRTEM image of 10.4 wt% Ni_12_P_5_/SiO_2_ composite.

Nickel phosphide materials were prepared by reducing nickel phosphate oxides under H_2_ flow. The XRD patterns, presented in Fig. 1a, showed that, in accordance with the Ni/P ratio of the chemical precursors, the as-prepared materials formed in the tetragonal Ni_12_P_5_ phase. In addition, a series of SiO_2_-supported Ni_12_P_5_ samples with different loadings (denoted by *x* wt% Ni_12_P_5_/SiO_2_, where the *x* is the weight percent of Ni_12_P_5_ determined by ICP-OES) were prepared via wet impregnation, with XRD patterns indicating that the composition and phase remained unchanged. The as-synthesized N_12_P_5_ exhibited a black color (Supplementary Fig. 1), and so diffuse reflectance UV-vis-NIR spectra were recorded to quantify its light absorption properties. As shown in Fig. 1b, the Ni_12_P_5_ nanoparticles exhibited broadband absorption throughout UV-vis-NIR region. Loading the Ni_12_P_5_ on SiO_2_, however, resulted in reduced optical absorption due to the dilution by the SiO_2_ support.

The morphology and crystalline structure of the samples were subsequently characterized by transmission electron microscopy (TEM). The low-magnification TEM image (Supplementary Fig. 2b) and the corresponding particle size distribution statistics (Supplementary Fig. 3a) showed that the diameters of unsupported Ni_12_P_5_ nanoparticles had a wide distribution with a mean diameter of 86 nm. Distinctive lattice fringe spacings of 0.604 nm and 0.401 nm, with an angle of 69°, were observed in the corresponding high-resolution TEM image (Fig. 1c), which is in accordance with the (110) and (101) planes of tetragonal Ni_12_P_5_. From the TEM images of Ni_12_P_5_/SiO_2_ samples (Supplementary Fig. 2c–e), mean particle diameters were distinctly smaller as the loading amount decreased, shrinking from about 13 nm in 10.4 wt% Ni_12_P_5_/SiO_2_ to about 8 nm in 3.1 wt% Ni_12_P_5_/SiO_2_ (Table 1). However, the high crystallinity was maintained, as shown by the HRTEM imaging on 10.4 wt% Ni_12_P_5_/SiO_2_ sample (Fig. 1d), and its tetragonal phase was again confirmed by the observation of lattice spacings corresponding to the (112) and (330) planes.

**Table 1:** Summary of properties and catalytic performances of representative Ni_12_P_5_ samples.

Sample	Ni_12_P_5_ TEM mean particle size (nm)	CO rate (mmol g_cat_^−1^ h^−1^)	CO selectivity (%)
Ni_12_P_5_	86 ± 30	156 ± 3	99.5 ± 0.1
10.4 wt% Ni_12_P_5_/SiO_2_	13 ± 7	960 ± 12	99.7 ± 0.1
5.2 wt% Ni_12_P_5_/SiO_2_	9 ± 3	678 ± 13	99.7 ± 0.1
3.1 wt% Ni_12_P_5_/SiO_2_	8 ± 4	334 ± 10	99.7 ± 0.1

X-ray photoelectron spectroscopy (XPS) was further performed to characterize the chemical states of the Ni_12_P_5_ surface. As shown in Supplementary Fig. 4a, the high-resolution Ni 2p region of the pristine Ni_12_P_5_ sample, before the photocatalytic reaction, consisted of two characteristic peaks located at 852.6 eV and 869.6 eV, which could be indexed to near zero-valent nickel. For the P 2p region (Supplementary Fig. 4b), two peaks were observed at 129.5 eV and 130.3 eV, and could be assigned to near zero-valent phosphorus. These values are in accordance with those reported in the literature^21,22^. Moreover, it is worth noting that the binding energy of Ni 2p_3/2_ is slightly higher than that of metallic Ni, while the P 2p_3/2_ binding energy is correspondingly lower than that of elemental P, thus indicating that Ni and P were partially positively and negatively charged, respectively, in Ni_12_P_5_.

### X-ray absorption spectroscopy measurements

**Fig. 2:**
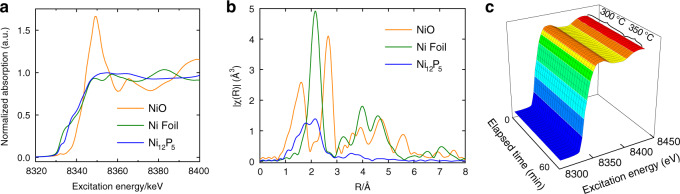
XAS measurements. **a** Ni K-edge X-ray absorption near-edge structure (XANES) spectra, with Ni Foil and NiO powder included as references. **b** R-space spectra. **c** Time-resolved in situ XANES curves plotted in the simulated reaction condition (CO_2_:H_2_ = 5:1 gas flow, with a temperature of 300 °C or 350 °C).

To further explore the structural features of Ni_12_P_5_, X-ray absorption spectroscopy measurements were conducted. As shown in the Ni K-edge X-ray absorption near-edge structure (XANES) spectra (Fig. 2a), the position and low intensity of the white line reveal the largely metallic character of Ni_12_P_5_, especially relative to the shifted and very intense peak observed for the strongly ionic NiO reference. These results thus provide very strong evidence supporting the low oxidation state of Ni atoms in Ni_12_P_5_. The bonding environment in Ni_12_P_5_ was further investigated using extended X-ray absorption fine structure (EXAFS) analysis. As shown in Fig. 2b, two coordination shells centered at 0.163 nm and 0.264 nm were observed in the Fourier-transformed Ni K-edge EXAFS spectra of Ni_12_P_5_, which resulted from Ni−P and Ni−Ni scattering paths, respectively. The dramatically reduced Ni–Ni bond peak in Ni_12_P_5_ relative to that of the Ni foil implied that the number of direct Ni–Ni bonds was reduced, due to the preferred formation of Ni–P bonds. Further curve-fitting results (Supplementary Table 1) indicated that Ni–Ni bond length was stretched from 2.480 Å in Ni foil to 2.510 Å in Ni_12_P_5_, and that the coordination number was significantly decreased from 12 in bulk Ni to 7 ± 1 in Ni_12_P_5,_ which is in accordance to its theoretical crystal structure^23^. Thus, these EXAFS fitting results strongly support the formation of well-dispersed, low-coordinate Ni nanoclusters with slight structural distortions relative to the bulk metal.

### Photocatalytic performance

Testing of photocatalytic CO_2_ hydrogenation by Ni_12_P_5_ catalysts was performed in a batch reactor, with samples loaded onto a borosilicate glass microfiber filter. The reaction was conducted under an initial total pressure of 18 psi (about 1.2 bar, CO_2_/H_2_ = 5:1), with a light intensity of 2.3 W cm^−2^ provided by an unfiltered Xe lamp. No external heating was adopted when using the batch reactor. Following completion of the photocatalytic reaction, products were quantified via gas chromatography. According to the GC traces (Supplementary Fig. 5), the product contained CO and a tiny amount of CH_4_, other products such as paraffin/olefin and methanol were not observed.

**Fig. 3:**
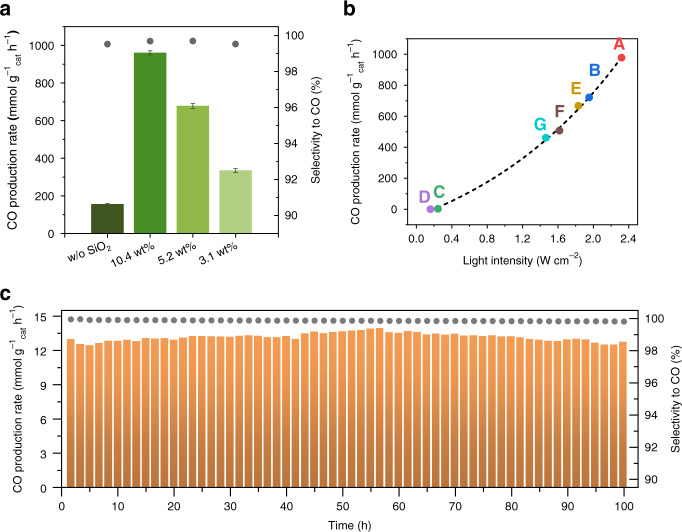
Light-driven RWGS reaction testing over Ni_12_P_5_ and Ni_12_P_5_/SiO_2_. **a** CO production rate and selectivity as a function of catalyst loading, tested in a batch reactor under 2.3 W cm^−2^ illumination without external heating, the initial reactant comprise 15 psi of CO_2_ and 3 psi of H_2_ (total pressure of 1.2 bar). Error bars are based on standard division. **b** CO production rates plotted as a function of light intensity as controlled by (A) full-spectrum light without any filter, (B) a neutral-density (ND) 0.1 filter, (C) a ND 0.3 filter, (D) a ND 0.5 filter, (E) a 420 nm cut-off filter, (F) a 495 nm cut-off filter and (G) a 590 nm cut-off filter, tested in a batch reactor. **c** Long-term stability testing over 10.4 wt% Ni_12_P_5_/SiO_2_ in a flow reactor, under both 0.8 W cm^−2^ light illumination and thermal activation, with 2.5 sccm of CO_2_ and 0.5 sccm of H_2_ gas flow.

As summarized in Table 1 and plotted in Fig. 3a, a selectivity of 99.5% towards the RWGS route was observed, with an average CO production rate of 156 ± 3 mmol g_cat_^−1^ h^−1^ being attained over unsupported Ni_12_P_5_ nanoparticles. Loading the Ni_12_P_5_ onto SiO_2_ further accelerated the photocatalytic reaction rate, and a maximum rate of 960 ± 12 mmol g_cat_^−1^ h^−1^ was achieved by the optimized 10.4 wt% Ni_12_P_5_/SiO_2_ sample. This corresponds to a 5.2-fold enhancement that is quite reasonable, given that the correspondingly smaller particle size would enlarge the specific surface area and expose more accessible reaction sites per gram of catalyst, as observed from the Ni dispersion results (Supplementary Note 1). However, further lowering the loading amount results in a decrease of the CO production rate along with the turnover frequency value (for calculation details see Supplementary Note 1). One plausible explanation is the inferior light harvesting ability on lower loading samples (Fig. 1b).

As summarized in Supplementary Table 2, this rate of CO_2_ hydrogenation to CO is a strong competitor to other reported photothermal catalysts, such as the commercial iron-chrome based catalyst, which delivered a CO production rate of 63.0 mmol g_cat_^−1^ h^−1^ under the same test conditions. It should also be emphasized that selectivity toward the RWGS reaction was unchanged for the SiO_2_-supported Ni_12_P_5_ samples, remaining at around 99.7%. Subsequent photocatalyst recyclability tests indicate that both the yield and selectivity were well retained over eight sequential runs (Supplementary Fig. 6), and XRD patterns from the spent sample confirmed that no Ni metal emerged following photocatalytic testing (Supplementary Fig. 7). XPS plots of the spent Ni_12_P_5_ sample following photocatalytic testing were also recorded and showcased identical binding energies for both Ni 2p and P 2p, confirming that the oxidation state of both Ni and P elements remained unchanged at the Ni_12_P_5_ surface. To more closely monitor catalyst stability under simulated RWGS reaction conditions, time-resolved in situ XANES tests were performed under a 1:5 (v/v) flow of H_2_/CO_2_ gas at 300 ^o^C over a 40 min period. Again, no significant changes were observed in the time-resolved XANES spectra (Fig. 2c), even after further increasing the temperature to 350 ^o^C for another 20 min, thus confirming the high stability of Ni_12_P_5_ in the RWGS reaction.

Isotope tracing experiments were further conducted by replacing the normal ^12^CO_2_ reagent gas with isotopically labeled ^13^CO_2_ and using gas chromatography–mass spectrometry (GC–MS) to analyze the chemical products. As shown in Supplementary Fig. 8, the signal corresponding to ^13^CO (*m*/*z* = 29) in the mass spectrum clearly confirmed that the product CO originated from the CO_2_ feedstock rather than any carbon contaminants.

To elucidate the role of light in the catalytic performance of Ni_12_P_5_, a series of photocatalytic tests was first conducted under various light intensities by employing a set of neutral-density filters and high-pass cut-off filters. As plotted in Fig. 3b, both visible and NIR light were able to activate the Ni_12_P_5_ catalyst and promote the RWGS reaction. The plot of CO production rate versus light intensity was well fitted by an exponential function, indicating a predominantly photothermal mechanism, where the catalyst absorbs and convert the incident photon flux into heat to drive the subsequent catalytic reactions^24^. This is distinct to the photochemical process in which the chemical reaction is driven directly by the photo-generated charge carriers, and the reaction rate would usually scale with the light intensity. Furthermore, as shown in Supplementary Note 2, the internal quantum yield (IQY) was estimated, indicating a trend that lower Ni_12_P_5_ loading samples delivered inferior IQY values. As mentioned, this could relate to the light harvesting ability as better light absorption would contribute to a higher local temperature under light stimulation, and thereof a superior TOF value. Nevertheless, the IQYs of Ni_12_P_5_ and 10.4 wt% Ni_12_P_5_/SiO_2_ are quite close. Considering only about one tenth of the active Ni_12_P_5_ has been utilized in the 10.4 wt% Ni_12_P_5_/SiO_2_ sample when compared to pristine Ni_12_P_5_, it is conceivable that the former would be more competitive overall, especially if the system was to be scaled-up. The photothermal effect on 10.4 wt% Ni_12_P_5_/SiO_2_ sample was further quantified by using ASPEN Plus software to estimate the local temperature of the catalyst surface based on the experimentally measured CO_2_ conversion at reaction equilibrium^4^. Under 2.3 W cm^−2^ irradiation and using a 5:1 ratio of CO_2_/H_2_, the photocatalytic reaction finally achieved 7.48% CO_2_ conversion in the batch reactor after 11.5-h test (Supplementary Fig. 9). The corresponding predicted local temperature approached 401 °C on a wet basis and 381 °C on dry basis (Supplementary Table 3), thereby demonstrating the exceptional photothermal capabilities of Ni_12_P_5_.

### Long-term stability evaluations

To highlight the excellent stability of Ni_12_P_5_, a 100-h long-term test was conducted in a flow reactor system in which the H_2_ and CO_2_ gases were continuously flowed through the catalyst and an external heat input was adopted. As shown in Fig. 3c, under a light irradiation with intensity of 0.8 W cm^−2^ and a controlled apparent temperature of 290 °C, the 10.4 wt% Ni_12_P_5_/SiO_2_ sample afforded an initial CO production rate and selectivity of 13.5 mmol g_cat_^−1^ h^−1^ and 99.9% during the first 10 h, respectively. respectively, and only a very small decrease to 13.3 mmol g_cat_^−1^ h^−1^ and 99.8% was observed after 100-h of continuous testing, thereby demonstrating its excellent long-term stability. In comparison, a control Ni/SiO_2_ sample lost 76% of its initial CO production rate after 100 h under similar conditions and declined to a final CO selectivity of about 85% (Supplementary Fig. 10).

In addition, in the same flow reactor tests were also performed at different temperatures with and without light illumination. As shown in Supplementary Fig. 11, under thermal activation (dark conditions), the 10.4 wt% Ni_12_P_5_/SiO_2_ sample can afford a CO production rate of 8.03 mmol g_cat_^−1^ h^−1^ at 320 °C. Furthermore, the CO production rate under light conditions (i.e., both thermal and photo activation) at the same apparent temperature was 25.6 mmol g_cat_^−1^ h^−1^, corresponding to a 2.2-fold enhancement. This enhancement was more significant at lower apparent temperature, which again indicated a solar advantage of the photothermal effect of the Ni_12_P_5_ catalyst.

The TEM images of the spent photocatalysts offered further evidence of the excellent anti-sintering ability of Ni_12_P_5_/SiO_2_, which was conducive to its remarkable stability. As shown in Supplementary Fig. 12, the low-magnification image and particle size distribution of the Ni_12_P_5_/SiO_2_ photocatalyst indicated that the overall mean particle size increased only slightly from about 13 nm to about 14 nm after 100 h of photocatalytic reaction. According to the corresponding HRTEM image, a high degree of crystallinity was well retained, implying the robust stability of the Ni_12_P_5_ catalyst, as well as its RWGS activity. In comparison, highly dispersed Ni metal nanoparticles of about 3.3 nm in diameter were found to have strikingly grown during long-term testing, which likely accounts for its considerable performance decay (Supplementary Fig. 13). The serious sintering of metallic Ni catalysts may be related to Mond process chemistry^25^, in which the Ni first reacted with the product CO to form the Ni(CO)_4_, and at higher temperature the Ni(CO)_4_ decomposes back to larger Ni particles. The formation of Ni(CO)_4_ was notably significantly suppressed on nickel phosphide^26,27^, thus reducing the possibility of Mond process and consequent sintering.

### In situ DRIFTS studies

**Fig. 4:**
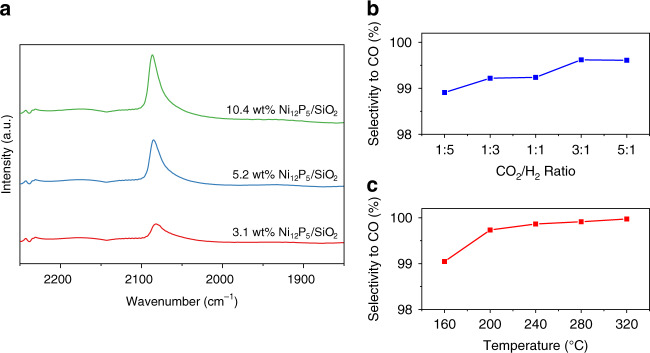
Determination of reaction pathway. **a** In situ DRIFTS spectra of Ni_12_P_5_/SiO_2_ samples with different loading amount. **b** The selectivity to the RWGS reaction over 10.4 wt% Ni_12_P_5_/SiO_2_ sample, plots with respect to the CO_2_/H_2_ initial ratio, tested in a batch reactor under 2.3 W cm^−2^ illumination without external heating. The initial total pressure was controlled as 18 psi (1.2 bar). **c** plots of the selectivity to the RWGS reaction over 10.4 wt% Ni_12_P_5_/SiO_2_ sample with respect to the temperature, tested in a flow reactor under 0.8 W cm^−2^ illumination and a gas flow of CO_2_ (2.5 sccm) and H_2_ (0.5 sccm).

**Fig. 5:**
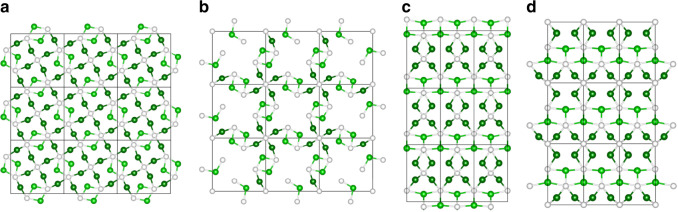
Surface crystal structure perspective of Ni_12_P_5_. **a**, **b** In the (001) orientation, **c**, **d** in (010) orientation. Note the white spheres represent the P atoms, and Ni atom with two different coordination environments are depicted as dark green and light green spheres, respectively.

In situ diffuse reflectance infrared Fourier transform spectroscopy (DRIFTS) measurements were conducted to investigate the reaction mechanism occurring at the Ni_12_P_5_ surface. DRIFTS spectra were acquired at 300 °C for 1 h in CO_2_/H_2_ flow after equilibrium, as presented in Fig. 4a. All DRIFT plots exhibited a pronounced peak centered at 2082 cm^−1^ and a weak, broad peak centered at 1920 cm^−1^, which could be assigned to linearly bonded Ni^0^-CO and bridge-bonded Ni^0^-CO, respectively^28,29^. The positions of the aforementioned two peaks are the same, regardless of the loading and size of the Ni_12_P_5_ particles, however, with a noticeable broadening and pronounced asymmetry of FTIR peak as the loading amount decreased, based on the FWHM analysis (Supplementary Table 4). This indicates that there is a range of surface sites for linearly bonded carbonyl species on the Ni_12_P_5_ particles, likely caused by the two distinct types of Ni site in Ni_12_P_5_ and their arrangements in different exposed facet^30,31^. Nevertheless, the intensity of the former peak overwhelms that of the latter, indicating that most carbonyl molecules preferred bonding individually to separated Ni atoms over engaging in multi-coordinate bonding. Regarding the metallic Ni on which the carbonyl molecule would be bonded in both linearly and bridging modes, observations based on the DRIFTS spectra of Ni_12_P_5_ implied the occurrence of unique, linearly bonded nickel-carbonyl-dominated reaction pathway. This can be explained by the ensemble effect^32–34^, as in this case the P atoms in Ni_12_P_5_ effectively separate Ni atoms into highly dispersed nanoclusters (as depicted in the crystal structure of the Ni_12_P_5_ in Fig. 5). As aforementioned, EXAFS results concluded that bonding between Ni and P resulted in an increased Ni–Ni bond length, which could geometrically hamper carbonyl bridge-bonding^35,36^. In addition, the absence of the fingerprint modes of formate species (as indicated in Supplementary Fig. 14) implied that the reverse water gas shift reaction over Ni_12_P_5_ likely occurred through the direct CO_2_ dissociation route^10^, in accordance with reported DFT predictions^37^.

The merit of linearly bonded carbonyl on Ni sites lies in the relative weaker binding energy when compared to those of bridge bonded species, and would facilitate the desorption of produced CO. In addition, the decreased coordination number of metal sites (i.e., Ni in Ni_12_P_5_) was deemed to interfere with the carbonyl dissociation and improved CO desorption^38^. Thereby, the consecutive reaction of CO with H_2_ for methane production would be hindered on the Ni_12_P_5_ surface, leading to a significantly near 100% selectivity to the RWGS reaction.

## Discussion

By virtue of the well-isolated Ni nanoclusters, the linearly bonded nickel-carbonyl-dominated reaction pathway along with high RWGS reaction rate and selectivity over Ni_12_P_5_ were well preserved when changing the reaction conditions. As shown in Fig. 4b and Fig. 4c, altering the initial CO_2_:H_2_ ratio (between 5:1 and 1:5) or reaction temperature (between 160 °C and 320 °C) had little influence on the selectivity. As a comparison, many studies have demonstrated that the reaction conditions, such as initial CO_2_/H_2_ ratio, CO_2_ conversion, temperature, catalyst particle size could significantly influence the CO_2_ hydrogenation selectivity over nickel related catalysts. For example, it has been reported that a higher proportion of H_2_ in the feed usually favors a high selectivity towards CH_4_ production^37^. Moreover, increasing the conversion of CO_2_ or raising the reaction temperature would also improve the selectivity to CH_4_ on a nickel catalyst^39,40^. The size of the nickel catalyst also play an important role in the reaction selectivity, where a smaller particle size would favor the formation of CO rather than CH_4_^10,40^. The size effect was more pronounced in the case of a Ni single atom catalyst, on which the CH_4_ production was almost eliminated^41^.

In stark contrast, the observations on Ni_12_P_5_ catalyst indicate that it can direct surface reaction pathways into the RWGS for CO production, and the near 100% CO selectivity for the Ni_12_P_5_ catalyst was virtually independent of overall particle size, gas composition and reaction temperature, which is a distinctive feature among nickel-containing catalyst. This unusual property thus enables Ni_12_P_5_ to function as a distinctive class of RWGS catalyst and can be considered to extend Ni catalyst science and technology towards broader applicability, such as in tandem with other catalysts known for their capacity for CO conversion reactions^42^.

Besides Ni_12_P_5_, the metal phosphide design concept was also demonstrated in the case of a cobalt phosphide analog. As shown in Supplementary Fig. 15, the as-prepared material can be well indexed to orthorhombic Co_2_P. Like Ni_12_P_5_, it is also able to absorb a wide range of the solar spectrum. In order to increase its specific area, the SiO_2_ supported Co_2_P was also synthesized, successfully shrinking the particle size to around 14 nm (Supplementary Fig. 16). Photocatalytic reactions over Co_2_P showcased a CO production rate of 15.7 mmol g_cat_^−1^ h^−1^ that was maintained for at least six usage cycles, and which could be further boosted to 227.7 mmol g_cat_^−1^ h^−1^ after loading onto SiO_2_ (Supplementary Fig. 17). It is worth noting that the selectivity of Co_2_P toward CO was still quite high (over 99%), and exhibited the same independence of overall particle size and CO_2_/H_2_ reactant ratio effects. Similar to Ni_12_P_5_, the crystalline structure of Co_2_P also displayed cobalt nanoclusters that were well-separated by P atoms (Supplementary Fig. 15d). Thus, the results presented herein over the Co_2_P photocatalyst have served to further validate the metal phosphide design philosophy.

In summary, the photothermal catalytic capability of Ni_12_P_5_ has been unlocked to drive a highly efficient reverse water gas shift reaction using simulated solar light, with a peak CO production rate of 960 ± 12 mmol g_cat_^−1^ h^−1^ and selectivity consistently greater than 99.5%. The key to this performance lies in the ensemble effect of P, which results in the highly dispersed Ni nanoclusters in Ni_12_P_5_, and disfavored strong multi-coordinate bonding to CO. In addition, the excellent light-harvesting ability of Ni_12_P_5_ across the entire solar spectrum also contributed to its photothermal activity, thereby enabling a considerably elevated local temperature to drive the RWGS reaction. Importantly, the advantages of high catalytic activity and light capture are not exclusive to Ni_12_P_5_, being shared by other transition metal phosphides such as Co_2_P. Therefore, as a class of high-performance, stable and cost-effective materials, the transition metal phosphides offer interesting opportunities for the development of photothermal CO_2_ conversion technologies.

## Methods

### Synthesis of Ni_12_P_5_ and SiO_2_-supported Ni_12_P_5_

A temperature programmed reduction (TPR) method was adopted to prepare the Ni_12_P_5_ nanoparticles. In detail, nickel nitrate and ammonium phosphate, (NH_4_)_2_HPO_4_, with molar ratio of 12:5 were first dissolved in DI water to form the precursor solution, and then dried at 120 °C, followed by annealing at 550 °C for 6 h to form nickel phosphate oxide. This precursor solid was then reduced under flowing 10 % H_2_/Ar at 600 °C for 6 h (with ramp of 10 °C min^−1^ from 25 °C to 300 °C and then 2 °C min^−1^ to 600 °C). The series of SiO_2_-supported Ni_12_P_5_ with various catalyst loadings was prepared via incipient wetness impregnation method. In a typical synthesize, the SiO_2_ was first immersed into a certain volume of the aforementioned precursor solution, and then followed by the same dry and H_2_-treatment process of the Ni_12_P_5_ synthesis. In addition, a Ni/SiO_2_ reference was also prepared in a similar impregnation route except the ammonium phosphate was not introduced into the precursor solution, and the H_2_-treament temperature was 350 °C.

### Material characterization

Powder X-ray diffraction patterns were recorded on a Bruker D2 Phaser X-ray diffractometer. UV-visible-NIR diffuse reflectance spectra were measured on Lambda 1050 UV/Vis/NIR spectrometer from Perkin Elmer, equipped with an integrating sphere. Low-resolution TEM images were taken on a FEI T12 G2 and high-resolution TEM images were taken on a Hitachi H-7650 HR-TEM. X-ray photoelectron spectroscopy (XPS) was performed on a Perkin Elmer Phi 5500 ESCA spectrometer in an ultra-high vacuum chamber, with all results being calibrated to the C 1*s* peaks at 284.5 eV. An Optima 7300DV ICP-OES apparatus (Perkin Elmer, USA) was used to measure the weight percent the Ni_12_P_5_ content in Ni_12_P_5_/SiO_2_ samples. Pulse H_2_ chemisorption was performed on AutoChem II 2920 to detect the metal dispersion on each sample, assuming atomic hydrogen only binds to surface nickel atoms with a Ni/H = 1.

X-ray absorption spectroscopy measurements were performed at Sector 9-BM of the Advanced Photon Source at Argonne National Laboratory (Lemont, IL) using a Quick-EXAFS monochromator and gas-ionization chamber detectors. A specially designed sample holder/chamber was used to provide simultaneous control of atmospheric composition and catalyst temperature during in situ measurements^43^. The Ni_12_P_5_ material was sealed in the sample chamber, purged with He and heated to 300 °C before flowing a 5:1 ratio of CO_2_/H_2_ and acquiring EXAFS spectra at 30 s intervals. XANES data processing was performed using Athena, part of the Demeter software package^44^. Fitting of EXAFS spectra was performed using WinXAS software^45^, in conjunction with scattering paths generated using FEFF8^46^ and based on crystal structures obtained from the Crystallographic Open Database^47^. The loading amount were calculated using ICP-AES data acquired on a Thermo iCAP 6300 spectrometer system.

### Photocatalytic gas-phase CO_2_ reduction tests

A batch reactor system was adopted to test the photocatalytic performance of the materials (detailed setup is shown in Supplementary Fig. 18). Before photocatalytic testing, 0.5 mg of the sample was well-dispersed in DI water via sonication and then drop-cast onto a binder-free borosilicate glass microfiber filter with an area of 1 cm^2^ before being dried under vacuum at 80 °C. The filter-supported sample was then fixed into a custom-fabricated 11.8 mL stainless steel batch reactor with a fused silica window and finally sealed with a Viton O-ring. The reactor was then evacuated before being sequentially filled with H_2_ and CO_2_ gas. The total pressure of the reactants was 18 psi (about 1.2 bar), with *P*(H_2_) = 3 psi and *P*(CO_2_) = 15 psi, as monitored by an Omega PX309 pressure transducer. An unfiltered 300 W Xe lamp was employed as the light source, and the light intensity was calibrated to 2.3 W with a light meter and a mask with an area of 1 cm^2^. No external heating was adopted in batch reactor. Photocatalytic reactions were conducted with a duration of 0.5 h for each run, after which the product gases were separated on an SRI-8610 gas chromatograph equipped with 30 Mole Sieve 13a and 60 Haysep D columns, and then analyzed using a flame ionization detector (FID) and thermal conductivity detector (TCD). For recyclable tests, the reactor was evacuated and refilled with the reactant gases after each run, without any treatments or air (oxygen) exposure. For tests under different light intensity and wavelength range, a series neutral-density (ND) filters and high pass cut-off filters were adopted, respectively. The ^13^C isotopic labeling experiments were also conducted in the same reactor, with the ^12^CO_2_ being replaced by ^13^CO_2_ and the products being measured on an Agilent 7890 A gas chromatograph/mass spectrometer equipped with a 60 m GS-CarbonPLOT column.

### Long-term photocatalytic gas-phase CO_2_ reduction tests

A flow reactor system was used for the long-term stability evaluation on the photocatalysts (detailed setup is shown in Supplementary Fig. 19). Firstly, the sample was packed into a tubular quartz capillary reactor with an inner diameter of 2 mm and an outer diameter of 3 mm. A gas flow of CO_2_ (2.5 sccm) and H_2_ (0.5 sccm) was then introduced into the reactor using Alicat Scientific digital flow controllers, and the temperature was regulated by an OMEGA CN616A temperature controller. The gas products were analyzed automatically on an SRI-8610, as described previously for photocatalytic activity measurements.

### In situ DRIFTS measurements

In situ DRIFTS experiments were conducted on a Thermo Scientific™ Nicolet™ iS50 FT-IR Spectrometer with a HgCdTe detector. A Harrick Praying Mantis™ diffuse reflection accessory and a Harrick high-temperature reaction chamber (HTC) with ZnSe windows were employed, with an additional Harrick ATC-024-3 controller used to provide temperature control. The samples were sealed into the sample cup and then heated to 350 °C under He flow (20 sccm) for 2 h. The samples were then cooled down to room temperature, during which time a series of background spectra were collected at different temperatures. Next, a mixture of CO_2_, H_2_ and He gases was purged into the chamber using flow rates of 5, 1 and 14 sccm, respectively. Finally, spectra were collected by taking 128 scans at each different temperature stage with a resolution of 4 cm^−1^.

### Synthesis of Co_2_P and SiO_2_-supported Co_2_P

The synthetic route used to produce Co_2_P is similar to that used for Ni_12_P_5_. Cobalt nitrate and (NH_4_)_2_HPO_4_ were first dissolved in DI water at a molar ratio of 2:1, then dried and calcined at 120 °C and 550 °C, respectively. These precursor solids were finally reduced in 10% H_2_/Ar flow at 720 °C for 6 h (with a ramp rate of 2 °C min^−1^ from 25 °C to 720 °C). Again, an incipient wetness impregnation route was adopted to load Co_2_P onto the SiO_2_ support material.

## Supplementary information

Supplementary Information

Peer Review File

## Data Availability

The data that support the plots within this paper and other findings of this study are available from the corresponding author upon reasonable request.
