# Supplementary material for: High-performance light-driven heterogeneous CO2 catalysis with near-unity selectivity on metal phosphides
Source: Nat Commun. 2020 Oct 13;11:5149. doi: 10.1038/s41467-020-18943-2 (PMC7555895; doi:10.1038/s41467-020-18943-2)
Supplement: Supplementary file 1 — Supplementary Information [file 41467_2020_18943_MOESM1_ESM.pdf]

Supplementary Information

High-Performance Light-Driven Heterogeneous CO_2 Catalysis with Near-Unity Selectivity on Metal Phosphides

Xu *et al.*

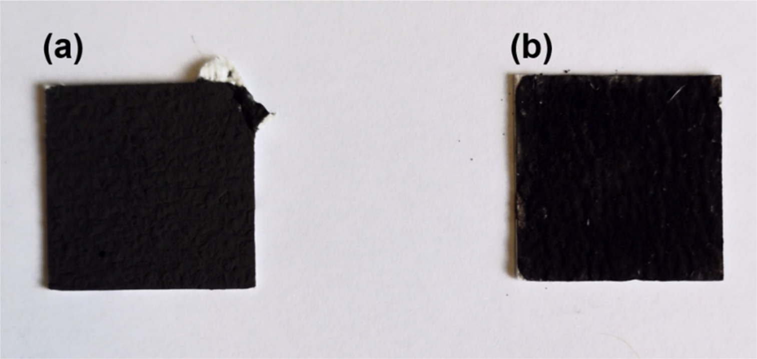

Supplementary Fig. 1 | Digital camera photographs. a. $Ni_{12}P_5$ and b. 10.4 wt% $Ni_{12}P_5/SiO_2$ samples loaded onto the borosilicate glass microfiber filter.

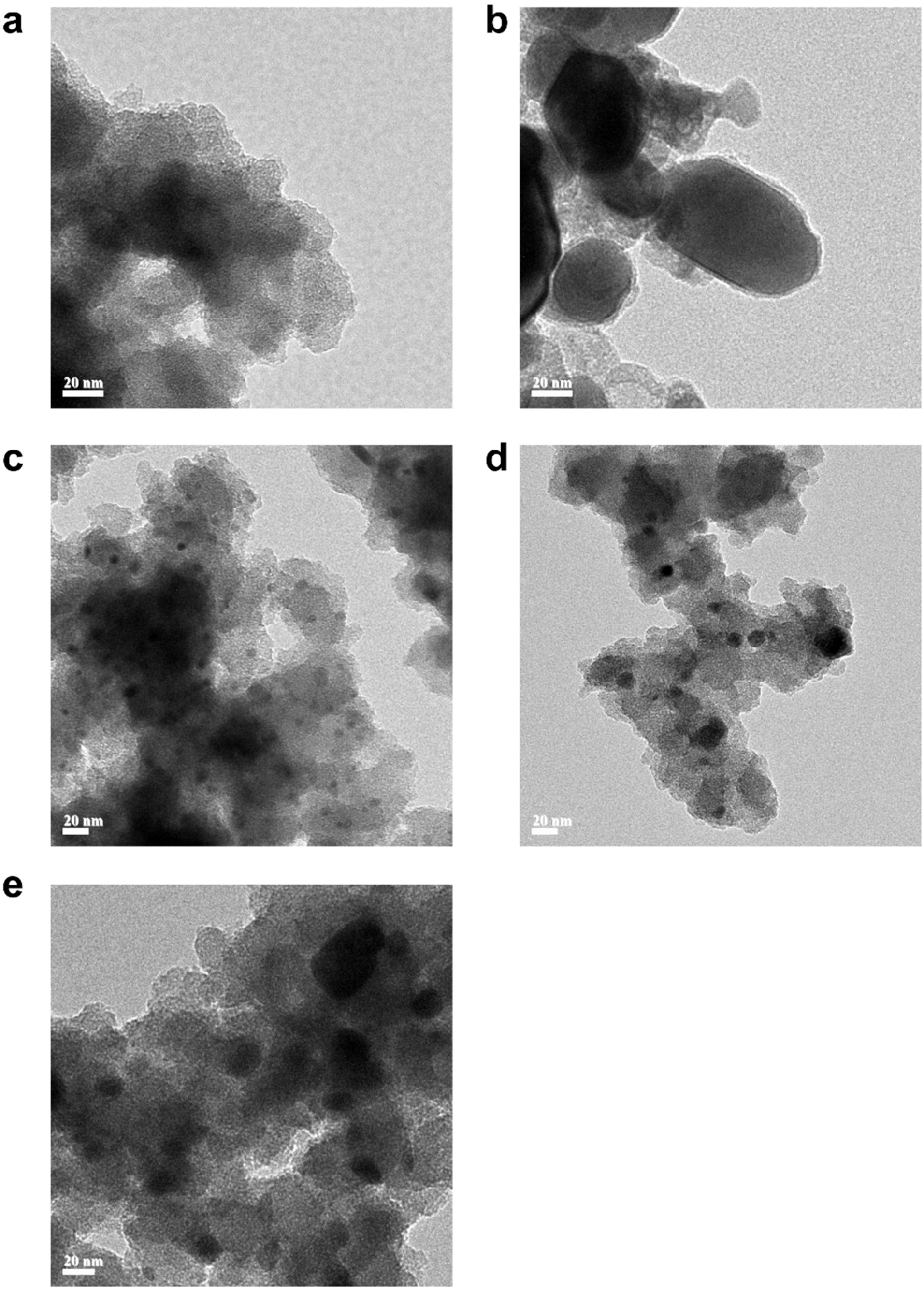

Supplementary Fig. 2 | Low-magnification TEM images of $Ni_{12}P_5$ and $Ni_{12}P_5/SiO_2$. a, SiO_2; b, $Ni_{12}P_5$; c, 3.1 wt% $Ni_{12}P_5/SiO_2$; d, 5.2 wt% $Ni_{12}P_5/SiO_2$; e, 10.4 wt% $Ni_{12}P_5/SiO_2$.

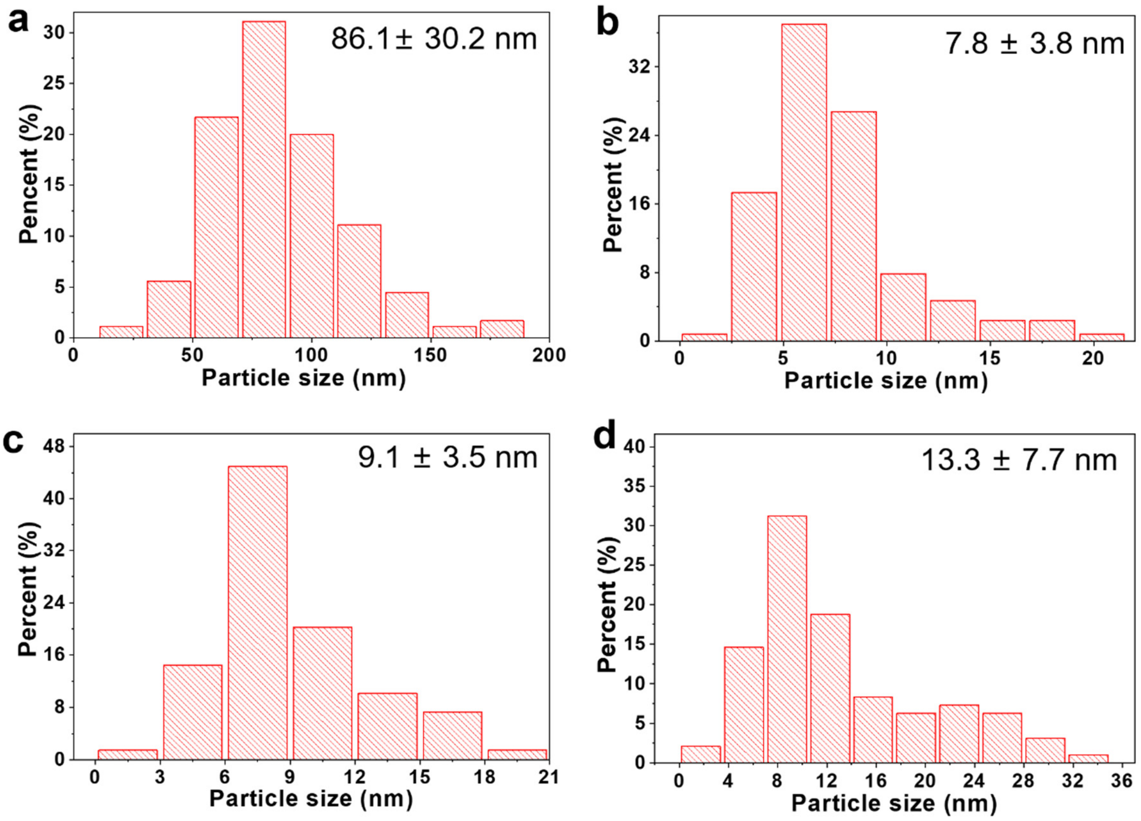

Supplementary Fig. 3 | Particle size distributions obtained from low-magnification TEM images. a, unsupported $Ni_{12}P_5$. **b,** 3.1 wt% $Ni_{12}P_5/SiO_2$. **c,** 5.2 wt% $Ni_{12}P_5/SiO_2$. **d,** 10.4 wt% $Ni_{12}P_5/SiO_2$.

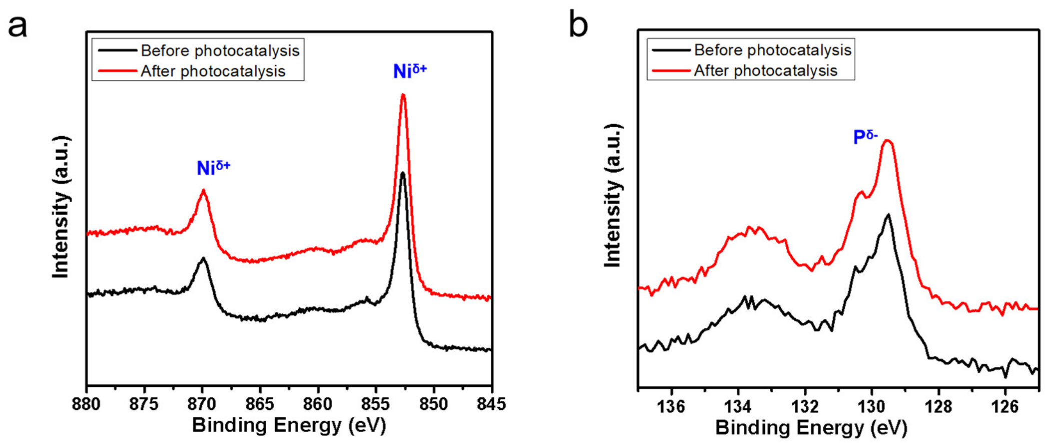

Supplementary Fig. 4 | High-resolution XPS plots of **a,** Ni 2p and **b,** P 2p regions before and after photocatalytic testing. Note a peak observed at 856.1 eV was attributed to the formation of oxidized Ni, while another at 133.6 eV was attributed to P in phosphate, suggesting that some partial surface oxidation occurred when exposing the sample to air.

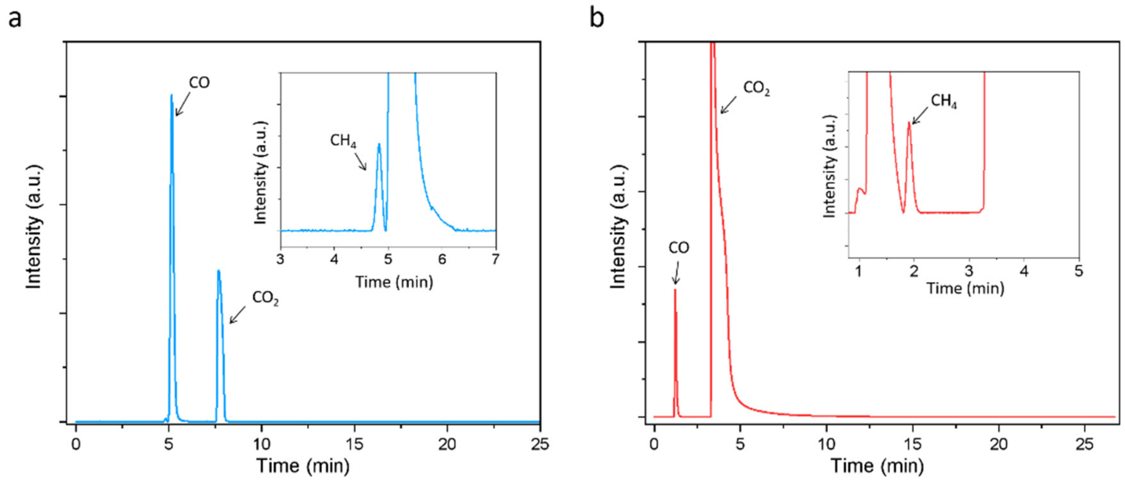

Supplementary Fig. 5 | The GC traces from catalyst testing in **a,** batch reactor and **b,** flow reactor. The insets are corresponding expanded view.

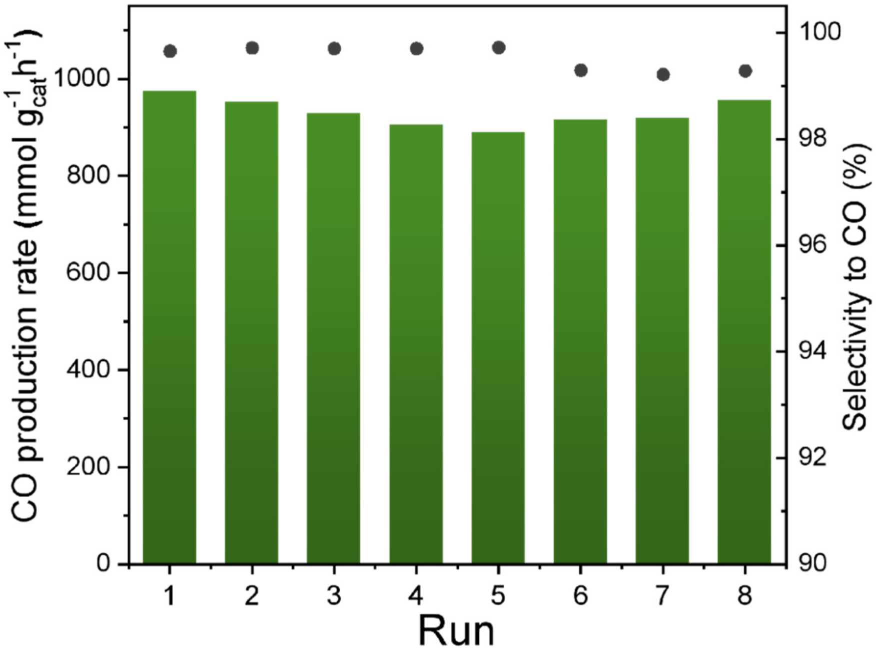

Supplementary Fig. 6 | Recyclability testing over the 10.4 wt% $Ni_{12}P_5/SiO_2$ sample in a batch reactor. The reactor was evacuated and refilled with the reactant gases after each run, without any treatments or air (oxygen) exposure.

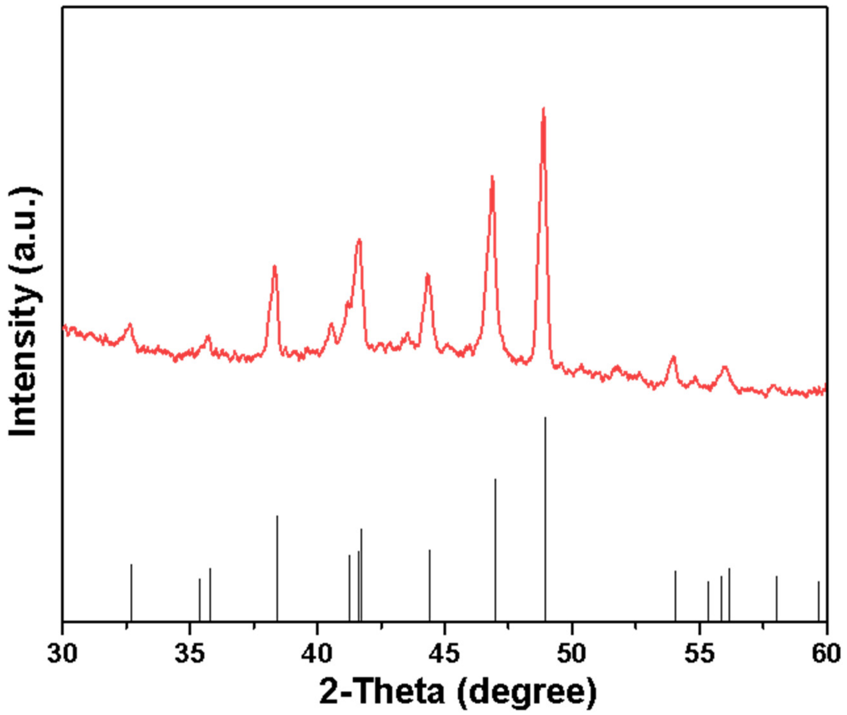

Supplementary Fig. 7 | PXRD patterns of the used $Ni_{12}P_5$ sample.

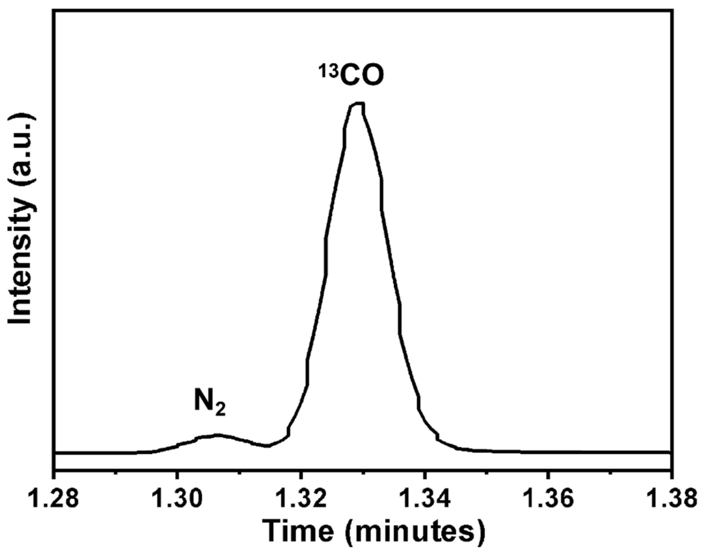

Supplementary Fig. 8 | GC-MS spectrum of the ^{13}C isotopic labeling experiment using $^{13}CO_2$ as the reagent gas.

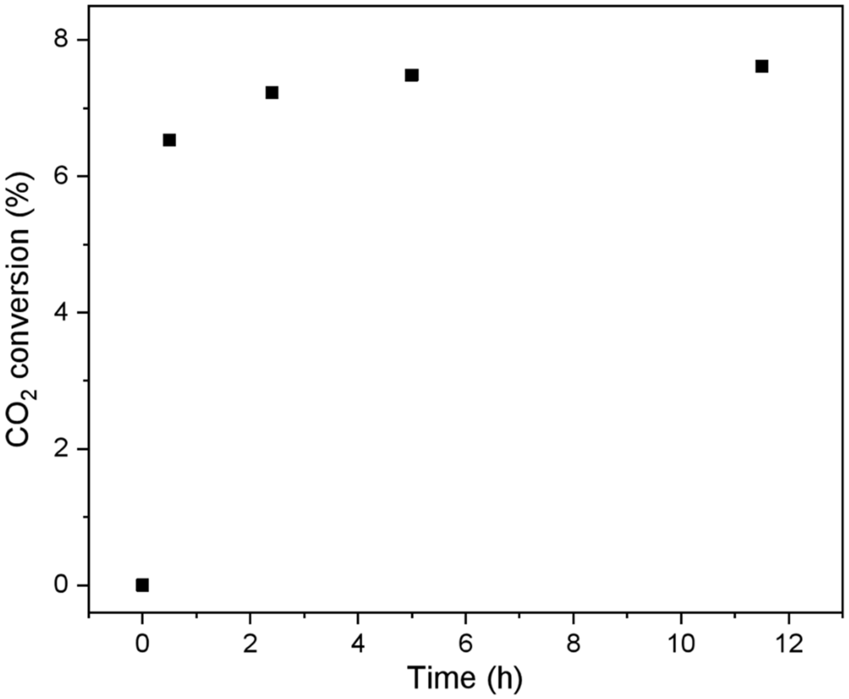

Supplementary Fig. 9 | Time course CO_2 conversion plots

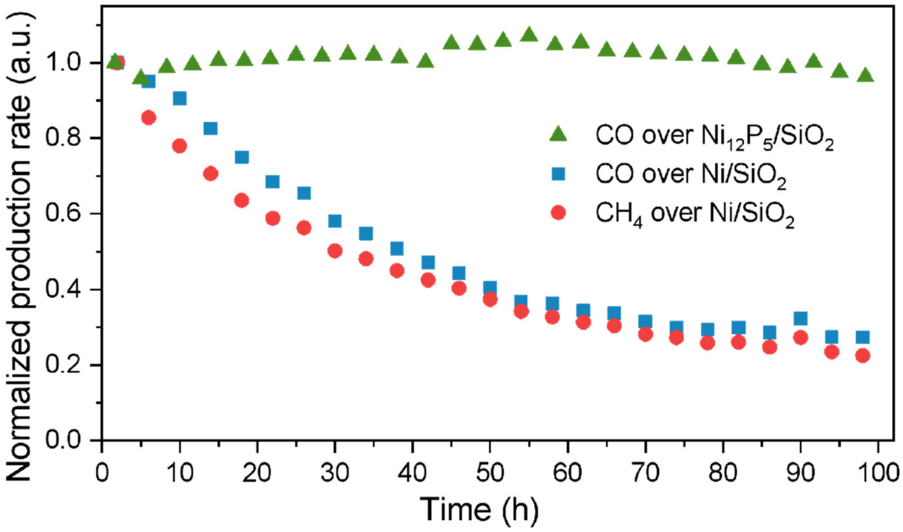

Supplementary Fig. 10 | Normalized production rates over the $Ni_{12}P_5/SiO_2$ and Ni/SiO_2 catalysts during the 100 h continuous test in the flow reactor system.

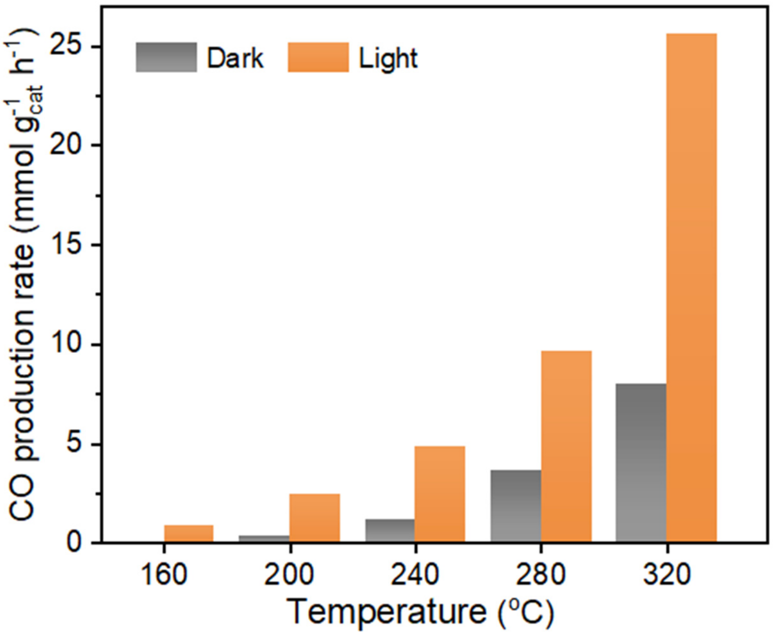

Supplementary Fig. 11 | Catalytic performance of 10.4 wt% $Ni_{12}P_5/SiO_2$. The tests were performed in a flow reactor at different reaction temperatures with and without solar irradiation. The gas flow contained 2.5 sccm of CO_2 and 0.5 sccm of H_2. The corresponding reaction selectivity data are shown in Fig. 4c.

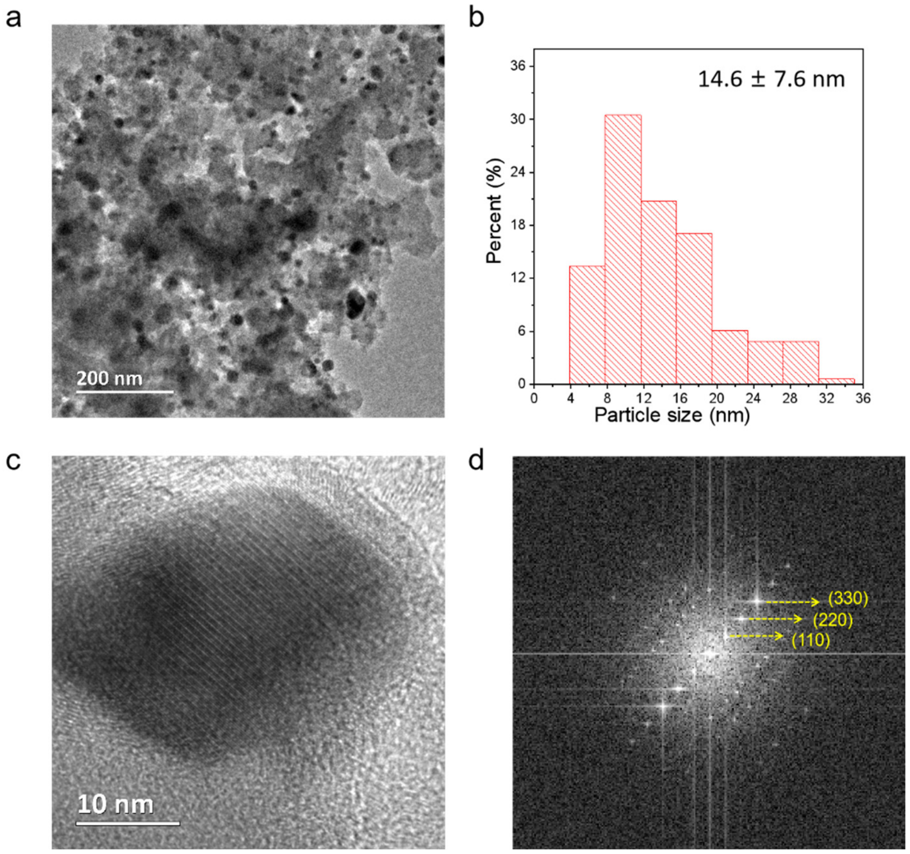

Supplementary Fig. 12 | Characterization of the $Ni_{12}P_5/SiO_2$ catalyst material after 100 h of continuous testing in the flow reactor system. a, Low-magnification TEM image. **b,** corresponding particle size distribution. **c,** high-resolution TEM image and **d,** corresponding FFT pattern.

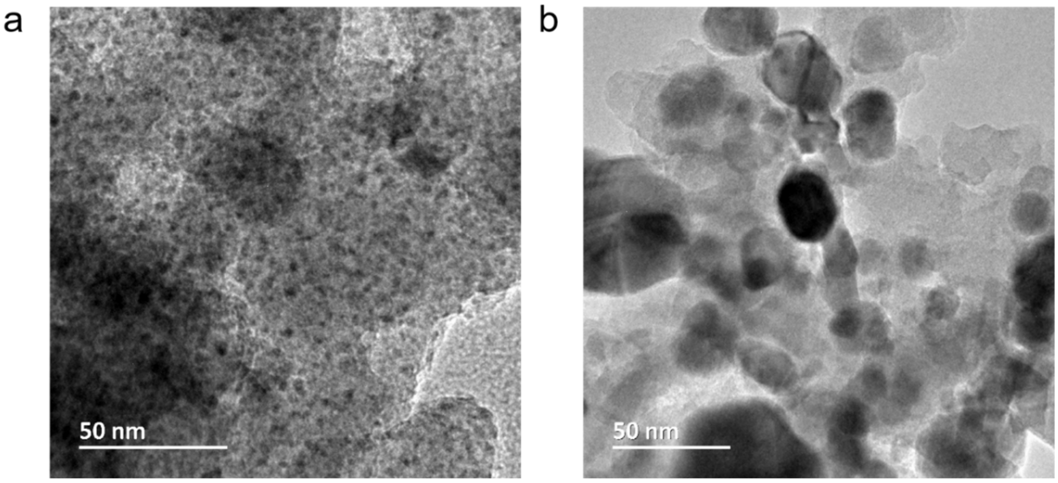

Supplementary Fig. 13 | TEM images of Ni/SiO_2 catalysts. a, fresh sample. **b,** used sample after 100 h continuous testing in the flow reactor system.

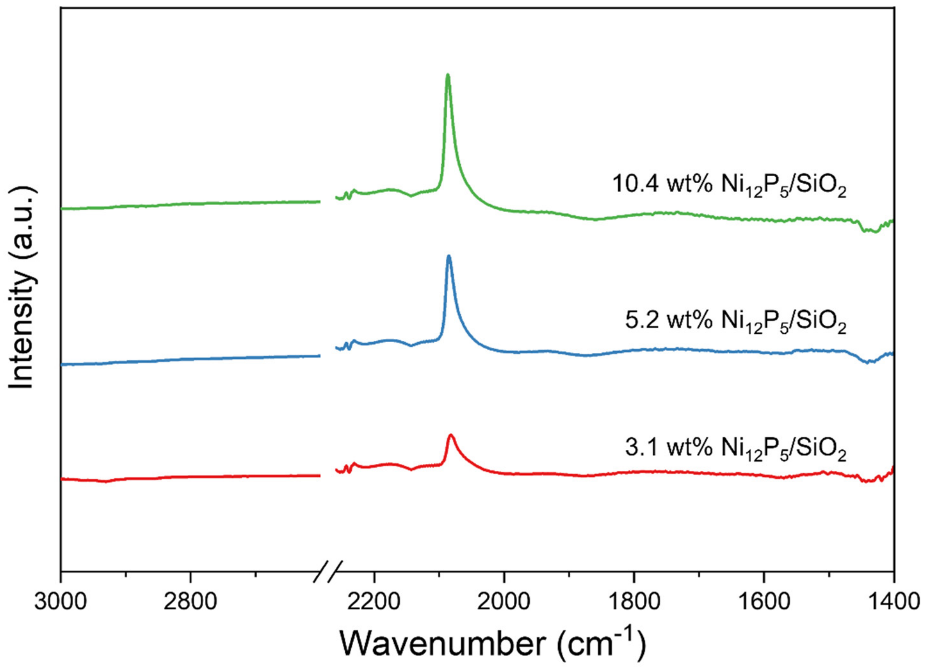

Supplementary Fig. 14 | In-situ DRITF survey spectra of $Ni_{12}P_5$-SiO_2 samples with different loading amount

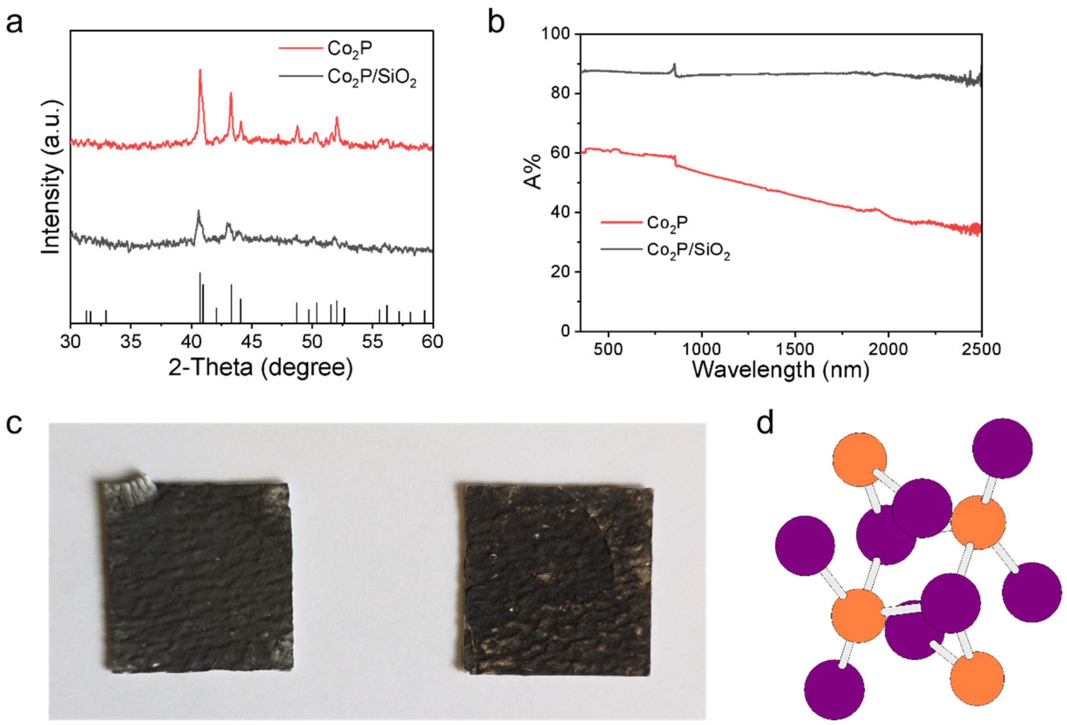

Supplementary Fig. 15 | Material characterizations of Co_2P. a, PXRD patterns and **b,** UV-vis absorption plots of as-prepared Co_2P. **c,** Digital camera photographs of the Co_2P (left) and Co_2P/SiO_2 (right) deposited onto borosilicate glass fiber filters. **d,** Crystal structure of Co_2P, purple atom is Co, orange atom is P.

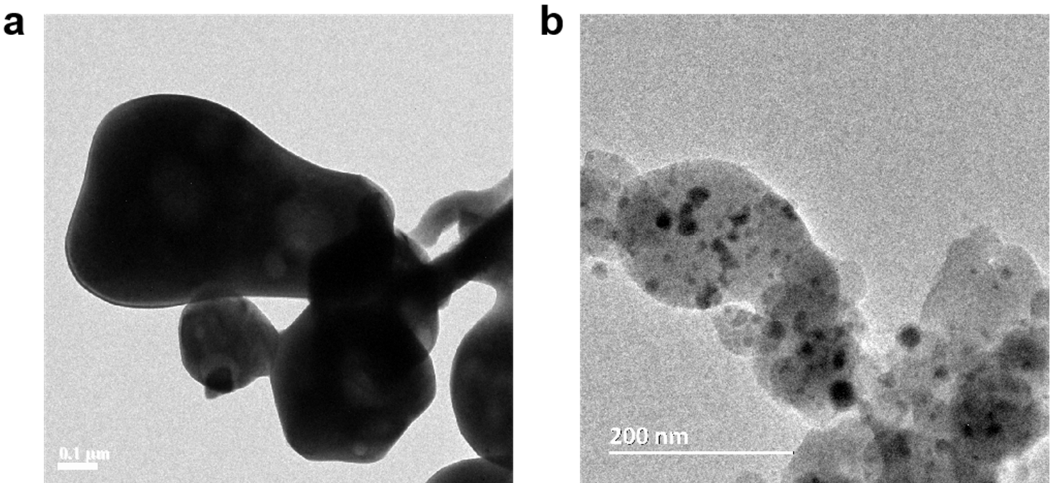

Supplementary Fig. 16 | TEM images of **a,** Co_2P and **b,** Co_2P/SiO_2 particles.

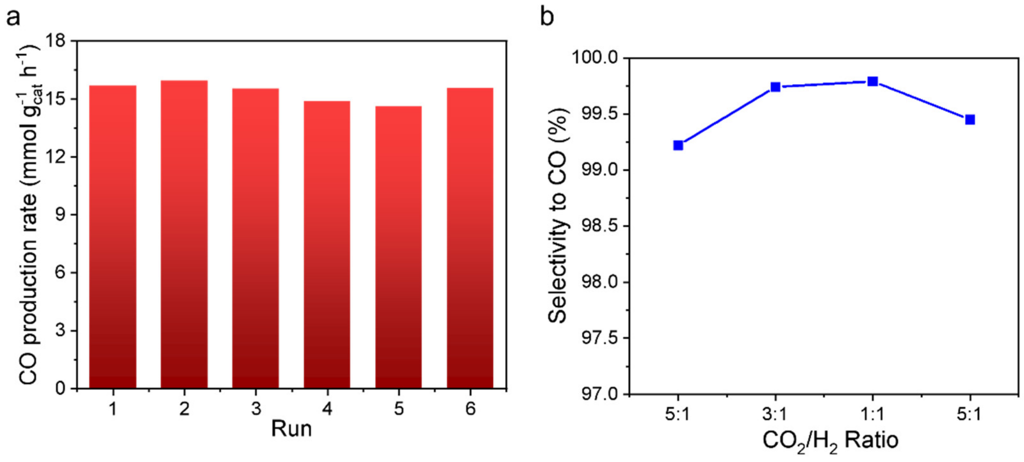

Supplementary Fig. 17 | Photocatalytic tests in a batch reactor over Co_2P. **a,** Recyclability testing via photocatalytic experiments over the Co_2P catalyst. **b,** selectivity plots with respect to the initial CO_2/H_2 ratio.

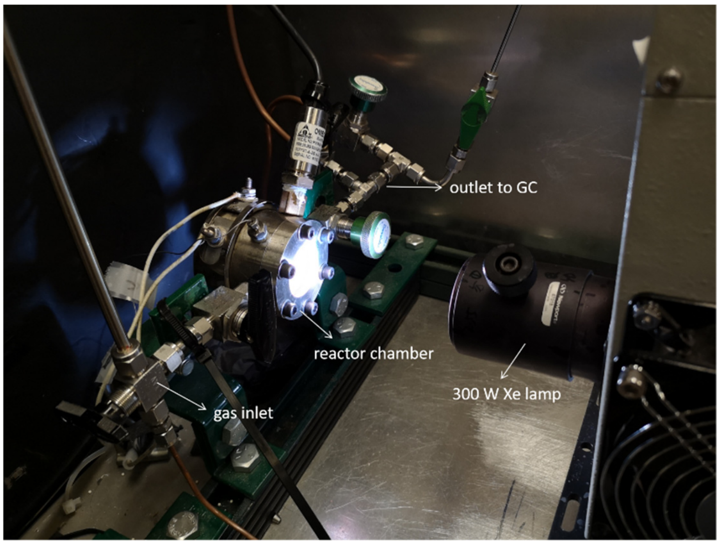

Supplementary Fig. 18 | Digital photograph of the batch reactor setup.

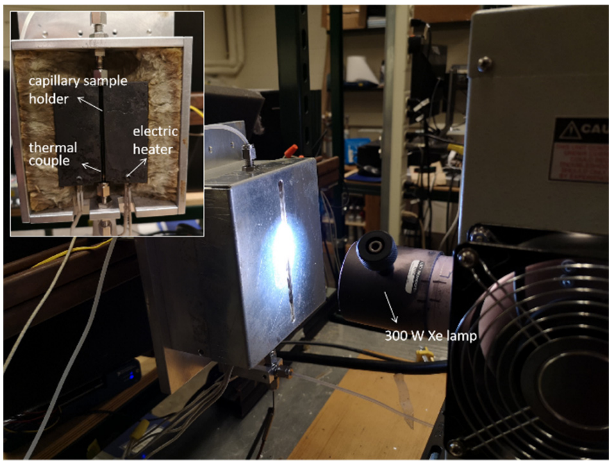

Supplementary Fig. 19 | Digital photograph of the flow reactor setup.

Supplementary Table 1. Curve Fitting Results of Ni K-Edge EXAFS of $Ni_{12}P_5$ Sample.

Sample	Scattering Path	CN (atoms)	Bond Length (Å)	Debye-Waller (10^{-3} Å^2)	E0 Shift (eV)
$Ni_{12}P5/SiO_2$ (25 °C)	Ni–P	3.9 ± 0.9	2.29 ± 0.01	21 ± 6	0 ± 1
	Ni–Ni	7 ± 1	2.510 ± 0.009	15 ± 2	

Supplementary Table 2. Comparison of CO_2 conversion rates for some CO_2 hydrogenation catalysts under light irradiation without external heating.

Catalyst	Light source	Feed composition	CO_2 conversion rate	Selectivity	
				CO	CH_4
Cu_2O[1]	300 W Xe light (full spectrum, 40 suns)	CO_2/H_2=83/17	70.3 mmol g^{-1} h^{-1}	~100	
In_2O_{3-x}[2]	300 W Xe light (~20 suns)	CO_2/H_2=50/50	238.8 mmol g^{-1} h^{-1}	~100	
$Pt/NaTaO_3$[3]	300 W UV-enhanced Xe lamp	CO_2/H_2=50/50	140.5 μmol g^{-1} h^{-1}	99	1
$Pd@Nb_2O_5$[4]	300 W Xe lamp	CO_2/H_2=50/50	1.8 m mol g^{-1} h^{-1}	100	
$Cu/Pd/HyWO_{3-x}$[5]	300 W Xe lamp (1 W cm^{-2})	CO_2/H_2=50/50	40.8 μmol g^{-1} h^{-1}	100	
Fe@C[6]	300 W Xe lamp	CO_2/H_2=50/50	26.1 mmol g^{-1} h^{-1}	100	
FeO–CeO_2[7]	300 W Xe lamp (2.2 W cm^{-2})	$CO_2/H_2/Ar$=15/60/25	20 mmol g^{-1} h^{-1}	97-99.9%	
SA Ni/Y_2O_3[8]	ambient daytime sunlight (from 0.52 to 0.7 kW m^{-2})	$CO_2/H_2/N_2$=2.5/10/87.5	7.5 L m^{-2} h^{-1}		100
$Ni/SiO_2{\cdot}Al_2O_3$[9]	solar simulator	$CO_2/H_2/N_2$=15/70/15	14.4 mmol g^{-1} h^{-1}	2.8	97.2
NiO[9]	solar simulator	$CO_2/H_2/N_2$=15/70/15	13.3 mmol g^{-1} h^{-1}		100
iron-chrome based catalyst† (this work)	300 W Xe lamp (2.3 W cm^{-2})	CO_2/H_2=83/17	63.0 mmol g^{-1} h^{-1}	99.9	0.1
$Ni_{12}P_5$ (this work)	300 W Xe lamp (2.3 W cm^{-2})	CO_2/H_2=83/17	155.7 mmol g^{-1} h^{-1}	99.5	0.5
$Ni_{12}P_5/SiO_2$ (this work)	300 W Xe lamp (2.3 W cm^{-2})	CO_2/H_2=83/17	960.3 mmol g^{-1} h^{-1}	99.7	0.3
Co_2P (this work)	300 W Xe lamp (2.3 W cm^{-2})	CO_2/H_2=83/17	15.7 mmol g^{-1} h^{-1}	99.2	0.8
Co_2P/SiO_2 (this work)	300 W Xe lamp (2.3 W cm^{-2})	CO_2/H_2=83/17	227.7 mmol g^{-1} h^{-1}	99.5	0.5

† Commercial iron-chrome based high temperature gas shift catalyst, HiFUEL® W210. The catalyst was milled before using.

Supplementary Table 3. Estimation of local temperature for $Ni_{12}P_5/SiO_2$ in the batch reactor system using ASPEN Plus, with the initial CO_2/H_2 ratio of 5:1 and irradiation intensity of 2.3 W cm^{-2}. Upon reaching reaction equilibrium, the conversion of CO_2 was 7.48%, corresponding to a CO concentration of 75,336.37 ppmv.

The accuracy of the ASPEN simulation depends on 1) the validity of the ideal gas approximation 2) if water stays in the gas phase for the duration of the experiment. Typically, we assume that 1) & 2) are met. 1) is valid for high temperature (>100 °C) and low-pressure systems (<2 atm).

For the estimation we used the ASPEN NRTL property package (assumes ideal gas phase) and the Gibbs reactor block that assumes all reaction between components (H_2, CO_2, CO, H_2O). A sweep was performed in 50 °C increments and the local temperature was determined by matching our GC results with the temperature dependent ASPEN output.

Wet basis mode (assuming product water reaches GC for detection):

Temp (°C)	CO_2 (ppmv)	CO (ppmv)	H_2 (ppmv)	H_2O (ppmv)	Sum (%)
**100**	827,018	5,982	161,018	5,982	100
**150**	820,373	12,627	154,373	12,627	100
**200**	810,777	22,223	144,777	22,223	100
**250**	798,768	34,233	132,767	34,233	100
**300**	785,289	47,711	119,289	47,711	100
**350**	771,377	61,622	105,378	61,622	100
**400**	757,914	75,086	91,914	75,086	100
**450**	745,505	87,495	79,505	87,495	100
**500**	734,478	98,522	68,478	98,522	100
**550**	724,936	108,064	58,936	108,064	100
**600**	716,827	116,173	50,827	116,173	100
**650**	710,018	122,982	44,018	122,982	100
**700**	704,339	128,661	38,339	128,661	100

Dry basis mode (assuming the water condensed in the lines before reaching the GC detector)

Temp (°C)	CO_2 (ppmv)	CO (ppmv)	H_2 (ppmv)	Sum (%)
**100**	831,995	6,018	161,987	100
**150**	830,864	12,788	156,347	100
**200**	829,204	22,728	148,068	100
**250**	827,081	35,446	137,473	100
**300**	824,633	50,101	125,266	100
**350**	822,033	65,669	112,298	100
**400**	819,443	81,182	99,376	100
**450**	816,987	95,884	87,128	100
**500**	814,749	109,289	75,962	100
**550**	812,767	121,157	66,076	100
**600**	811,049	131,443	57,508	100
**650**	809,582	140,227	50,190	100
**700**	808,341	147,659	44,000	100

Supplementary Table 4. The FWHM estimation of the FTIR peaks of linear bonded Ni-CO species.

Sample	FWHM (nm)
10.4 wt% $Ni_{12}P_5/SiO_2$	13.1
5.2 wt% $Ni_{12}P_5/SiO_2$	13.9
3.1 wt% $Ni_{12}P_5/SiO_2$	17.2

Supplementary Note 1. Details of TOF calculation

The TOF is calculated in terms of per nickel metal site, according to the following equation:

$$TOF(s^{-1}) = \frac{\text{Total CO turnovers per second}}{\text{Total Ni site numbers}}$$

Where the total CO turnovers per second is calculated as

$$\text{Total CO turnovers per second} = \frac{\mathrm{r} \times \mathrm{mass_{catalyst}}}{3600} = \frac{\mathrm{r} \times \mathrm{mass_{total}} \times x \times N_A}{3600}$$

Where r is the CO production rate (mmol g_{cat}^{-1} h^{-1}) presented in Table 1, $mass_{total}$ is the mass of the used catalyst (including $Ni_{12}P_5$ and SiO_2 supports), x is the weight percent of $Ni_{12}P_5$ in the $Ni_{12}P_5/SiO_2$, N_A is the Avogadro number.

To determine Ni site numbers, there are two methods:

Method 1: Estimate from metal dispersion result via H_2 adsorption measurement

$$\text{Total Ni site numbers} = \frac{\mathrm{mass_{total}} \times y \times D \times N_A}{\mathrm{M}}$$

where y is the weight percent of Ni in the sample, as determined from ICP-OES, D is the metal dispersion which was obtained from a pulse H_2 chemisorption experiment. Note the metal dispersion herein is determined with respect to the Ni metal, and assume atomic hydrogen only binds to surface nickel atoms with a H:Ni stoichiometry of 1.[10-11] M is the atomic weight of Ni, which is 58.69 g mol^{-1}.

Therefore, the TOF can be calculated according to the following equation:

$$TOF(s^{-1}) = \frac{\mathrm{r} \times \mathrm{mass_{total}} \times x \times N_A}{3600} \div \frac{\mathrm{mass_{total}} \times y \times D \times N_A}{\mathrm{M}} = \frac{r \times x \times \mathrm{M}}{3600 \times y \times D}$$

The results are listed in the following Table

Sample	Metal dispersion (%)	TOF (s^{-1})
$Ni_{12}P_5$	0.014	23.25
10.4 wt% $Ni_{12}P_5/SiO_2$	0.094	20.45
5.2 wt% $Ni_{12}P_5/SiO_2$	0.35	3.89
3.1 wt% $Ni_{12}P_5/SiO_2$	0.88	0.76

Method 2: Estimate from lattice structure

In this method the theoretical metal site concentration, L, was estimate based on the crystal structure of $Ni_{12}P_5$, according to the work published by Oyama et. al.[12] The theoretical metal site concentration assumes that the samples are composed of uniform spherical particles. This L is calculated by

$$L = S \times n$$

where n is the average surface metal atom density, and S is the effective surface area.

The effective surface area was calculated as

$$S = \frac{6}{\rho \times \mathrm{D}}$$

where ρ is the material density (7.53 g cm^{-3} for $Ni_{12}P_5$), D is the average particle size extract from the size distribution statistics based on TEM images (as shown in Table 1 and Supplementary Fig. 3).

Average surface metal atom density is estimated via a published method.[3] For the $Ni_{12}P_5$ there are six, six, and six Ni atoms on the ac, ab, and bc unit cell faces, respectively (Supplementary Fig. 20). The parameters are list in the following table.

**Lattice parameter (nm)**			**Surface metal density (10^{15} atoms cm^{-2})**			
a	b	c	ac	ab	bc	average
0.8629	0.8629	0.5036	1.38	0.805	1.38	1.19

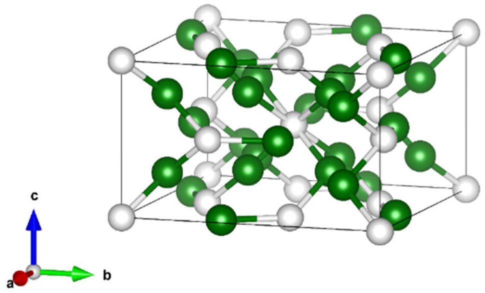

Supplementary Fig. 20 | Crystal structure of $Ni_{12}P_5$.

Therefore, the L and TOF was calculated as

**Sample**	**L (μmol g^{-1})**	**TOF (s^{-1})**
$Ni_{12}P_5$	183.0	0.24
10.4 wt% $Ni_{12}P_5/SiO_2$	1210.6	0.22
5.2 wt% $Ni_{12}P_5/SiO_2$	1748.7	0.11
3.1 wt% $Ni_{12}P_5/SiO_2$	1967.3	0.047

Supplementary Note 2. The calculation of the internal quantum yield of CO (IQY_{CO})

In this study the internal quantum yield was calculated as it can exclude the light absorption variations to make a fair comparison. The internal quantum yield of CO was defined as[13]

$$\text{Internal quantum yield}_{CO} = \frac{\text{produced CO molecules per unit time}}{\text{absorbed photon numbers per unit time}}$$

Where the absorbed photon numbers per unit time, N_{photon}, is estimated from the light intensity dispersion of the Xe lamp (Supplementary Fig. 21) and the UV-vis-NIR absorption spectra.

$$N_{photon} = \int_{300\,nm}^{2400\,nm} \frac{\text{Light intensity} * \text{ I\%} * \text{A\%} * \text{illmination area} * \text{ time}}{\text{Average single photon energy} * \ N_A}$$

Where the light intensity is 2.3 W, illumination area is 1 cm^2, I% is the percentage of the Xe light intensity at certain wavelength, A% is the light harvesting efficiency at certain wavelength according to the absorption spectra (Fig. 1b in manuscript), time is 3600s, N_A is the Avogadro constant. The average single photon energy (E_{photon}) is figured out using the equation:

$$\mathrm{E_{photon}} = \frac{hc}{\lambda}$$

where h is the Planck constant, c indicates speed of light, and λ is the wavelength.

Thus, we can figure out that total incident photons flux from the lamp is about 5.49 mmol/h, while for the $Ni_{12}P_5$, 10.4 wt% $Ni_{12}P_5/SiO_2$, 5.2 wt% $Ni_{12}P_5/SiO_2$, 3.1 wt% $Ni_{12}P_5/SiO_2$ samples they can absorb 92.3%, 73.2%, 55.5%, 47.7% of the photon flux, respectively. The internal quantum yield results are listed in the following table.

Sample	IQY_{CO}%
$Ni_{12}P_5$	0.060
10.4 wt% $Ni_{12}P_5/SiO_2$	0.059
5.2 wt% $Ni_{12}P_5/SiO_2$	0.026
3.1 wt% $Ni_{12}P_5/SiO_2$	0.0086

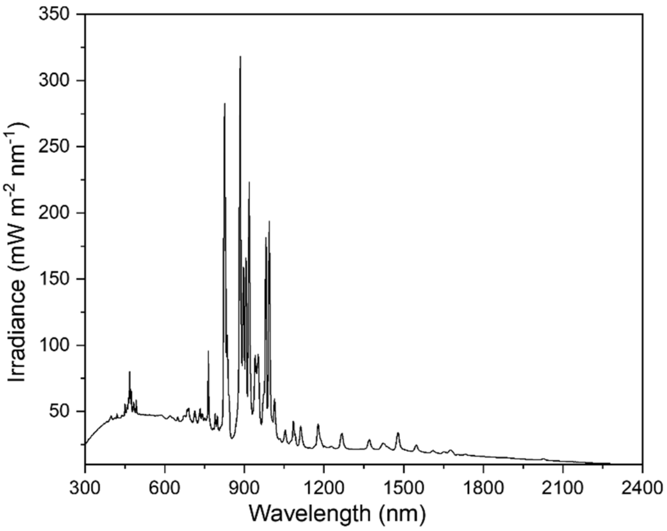

Supplementary Fig. 21 | Spectra of the 300 W Xe lamp.

Supplementary References

1 Wan, L. L. *et al.* Cu_2O nanocubes with mixed oxidation-state facets for (photo)catalytic hydrogenation of carbon dioxide. *Nat. Catal.* **2**, 889-898 (2019).

2 Wang, L. *et al.* Black indium oxide a photothermal CO_2 hydrogenation catalyst. *Nat. Commun.* **11**, 2432 (2020).

3 Li, M. *et al.* Highly efficient and stable photocatalytic reduction of CO_2 to CH_4 over Ru loaded NaTaO3. *Chem. Commun.* **51**, 7645-7648 (2015).

4 Jia, J. *et al.* Photothermal Catalyst Engineering: Hydrogenation of Gaseous CO_2 with High Activity and Tailored Selectivity. *Adv. Sci.* **4**, 1700252 (2017).

5 Li, Y. F. *et al.* Cu Atoms on Nanowire Pd/H_yWO_{3-x} Bronzes Enhance the Solar Reverse Water Gas Shift Reaction. *J. Am. Chem. Soc.* **141**, 14991-14996 (2019).

6 Zhang, H. *et al.* Surface-Plasmon-Enhanced Photodriven CO_2 Reduction Catalyzed by Metal-Organic-Framework-Derived Iron Nanoparticles Encapsulated by Ultrathin Carbon Layers. *Adv. Mater.* **28**, 3703-3710 (2016).

7 Zhao, J. *et al.* FeO–CeO_2 nanocomposites: an efficient and highly selective catalyst system for photothermal CO_2 reduction to CO. *NPG Asia Mater.* **12**, 5 (2020).

8 Li, Y. *et al.* Selective light absorber-assisted single nickel atom catalysts for ambient sunlight-driven CO_2 methanation. *Nat. Commun.* **10**, 2359 (2019).

9 Sastre, F., Puga, A. V., Liu, L., Corma, A. & García, H. Complete Photocatalytic Reduction of CO_2 to Methane by H_2 under Solar Light Irradiation. *J. Am. Chem. Soc.* **136**, 6798-6801 (2014).

10 Wojcieszak, R. *et al.* Nickel containing MCM-41 and AlMCM-41 mesoporous molecular sieves: Characteristics and activity in the hydrogenation of benzene. *Appl. Catal., A* **268**, 241-253 (2004).

11 Berenguer, A. *et al.* Catalytic hydrodeoxygenation of m-cresol over Ni_2P/hierarchical ZSM-5. *Catal. Today* **304**, 72-79 (2018).

12 Wang, X., Clark, P. & Oyama, S. T. Synthesis, Characterization, and Hydrotreating Activity of Several Iron Group Transition Metal Phosphides. *J. Catal.* **208**, 321-331 (2002).

13 Qureshi, M. & Takanabe, K. Insights on Measuring and Reporting Heterogeneous Photocatalysis: Efficiency Definitions and Setup Examples. *Chem. Mater.* **29**, 158-167 (2017).
